# Trends in smoking-related diseases by socioeconomic position following a national smoking ban in 2007: a nationwide study in the Danish population

**DOI:** 10.1186/s12889-023-16456-3

**Published:** 2023-08-28

**Authors:** Nanna Schneekloth Jarlstrup, Lau C. Thygesen, Charlotta Pisinger, Jørgen Vestbo, Morten Grønbæk, Janne S. Tolstrup

**Affiliations:** 1 National Institute of Public Health, University of Southern Denmark, Studiestræde 6, 1455 Copenhagen, Denmark; 2 Center for Clinical Research and Prevention, Bispebjerg and Frederiksberg Hospital, Capital Region, Copenhagen, Denmark; 3 University of Manchester and Manchester University NHS Foundation Trust, Manchester, UK

**Keywords:** Tobacco, Smoking ban, Smoking-related diseases, Socioeconomic position, Interrupted time series analysis

## Abstract

**Background:**

National comprehensive smoke-free legislation has been found to decrease the incidence of several smoking-related diseases. In 2007, Denmark introduced a national smoking ban, which banned smoking indoor in workplaces and public places, although only partial restrictions were applied in certain settings. We examined the impact of the smoking ban on smoking-related diseases and whether this differed across socioeconomic groups.

**Methods:**

Interrupted time series analyses of nationwide register data were performed using Poisson regression models to examine the differential impact of the smoking ban on monthly incidence rates of acute myocardial infarction, chronic obstructive pulmonary disease, and smoking-related cancers from 2002 to 2015. Immediate changes in incidence rates after the smoking ban and long-term changes in disease trends were estimated by comparing data from the pre- and post-ban period. Models were stratified by socioeconomic position.

**Results:**

Overall, we found neither immediate changes in rates of acute myocardial infarction, chronic obstructive pulmonary disease, and smoking-related cancers following the smoking ban nor long-term post-ban changes in disease trends as compared to before the ban. Results did not differ across socioeconomic groups. A pronounced socioeconomic gradient in incidence rates was observed for all outcomes both before and after the smoking ban.

**Conclusion:**

The national smoking ban was not associated with a lower incidence of smoking-related diseases in the post-ban period compared to pre-ban levels and no differences between socioeconomic groups were observed. Future tobacco control in Denmark should consider which measures most effectively target the low socioeconomic groups to decrease the current strong socioeconomic inequality in health.

## Background

Smoking is the single most important preventable cause of morbidity and premature mortality worldwide and is one of the most substantial global public health concerns [1]. Guided by international evidence of the negative health impacts of smoking and second-hand smoke (SHS) exposure, many countries have introduced national legislation to discourage smoking initiation, increase smoking cessation, and reduce SHS exposure [2, 3].

An important approach is the enactment of national smoking bans that restrict smoking in designated areas, including workplaces and public settings. The evidence is strong that comprehensive smoking bans are high-impact public health policies leading to reductions in adverse health outcomes in the general population [4, 5]. Previous studies in this field have mainly examined post-ban effects at population level [4, 6]. As any health benefits following a smoking ban may not be equivalent across population subgroups that differ in risk, incidence and burden of smoking-related morbidity, attention must also be given to the potential differential effect of smoking bans to prevent a widening of existing health disparities [6–8].

In most developed countries, smoking prevalence are particularly high in socioeconomic disadvantaged groups contributing to poorer health and higher mortality among those with lower socioeconomic position [9]. This manifests itself in a marked socioeconomic gradient in life expectancy [9, 10]. In Denmark, smoking displays large disparities and is strongly associated with lower socioeconomic position [11]. Further, smoking is one of the leading factors responsible for the socioeconomic inequality in health in the Danish population and smoking and alcohol have been identified as the main explanations for an increase in inequality in mortality observed from 1985 to 2009 [11, 12]. Although the smoking prevalence in the general population has decreased since 1970 to 19% in 2022, this has occurred at a slower pace among the lowest educated, thus increasing inequalities in smoking [13, 14]. In 2022, 22% of people with a primary school as the highest educational level were smoking daily in Denmark compared to only 8% among those with an advanced education [15]. This calls for tobacco control measures that strive to be effective in all socioeconomic groups.

In 2007, a national smoking ban was implemented prohibiting indoor smoking in public settings and workplaces. The ban was the most extensive tobacco control policy implemented at national level in Denmark at the time; however, several exemptions were made, resulting in only a partial coverage in specific premises such as small bars, one-man offices, and commercial vehicles [16]. The primary aim of the smoking ban was to protect non-smokers from the harmful effect of SHS exposure. Additionally, a reduction in population smoking prevalence and smoking intensity was anticipated.

While most previous studies have been examining the potential contribution of smoking bans to reduce socioeconomic inequalities in smoking behavior such as prevalence, cessation rates, and smoking intensity [1, 6, 17–19], less attention has been directed towards the differential impact on health outcomes. Existing research indicates greater post-ban reductions in mortality and hospital admissions for acute coronary events and chronic obstructive pulmonary disease (COPD) in socioeconomic disadvantaged groups [7], and in more deprived areas [20–22] compared with their socioeconomic counterparts. However, the evidence is limited, and findings are inconclusive. In this study we investigate whether the incidence of acute myocardial infarction (AMI), COPD, and smoking-related cancers differed across socioeconomic groups after the implementation of the Danish national smoking ban in 2007 compared to pre-ban levels.

## Methods

This study is based on data from nationwide population-based registers. Separately for each outcome, a study population consisting of the entire Danish adult population (30 + years) from 2000 to 2015 was linked with individual outcome records from the Danish National Patient Register, which collects admission records from Danish hospitals since 1977 as well as outpatient and emergency room contacts since 1995 [23]. As we examined smoking-related diseases predominantly occurring in the adult population, the study population was restricted to include individuals over 30 years of age. Incident cases of disease occurring during the study period (primary or secondary diagnosis) were identified, and pre-hospital deaths were accounted for by retrieving cause-specific mortality data from the Danish Register of Causes of Death (underlying or contributory cause of death) [24]. Diagnoses were classified according to the International Classification of Disease, tenth revision (ICD10) (ICD8: 1977–1993). Through the Civil Registration System, we obtained information on sex and age [25]. Data was obtained at individual level and linked within and across years through the unique personal identification number (CPR), which residents in Denmark are legally required to be assigned at birth or immigration [25]. A study population were defined for each outcome separately and included individuals without a preceding diagnosis of the outcome in question, who were Danish residents 1 January 2002, at their 30 years birthday, or at immigration, whichever came last. Disease event, death of any cause, emigration, disappearance, or end of the study period whichever came first marked the end of follow up. Individuals were continuously included in the study population during the study period if they were eligible for inclusion. No re-entry was allowed. The study population was divided in age- and calendar-specific strata and subsequently aggregated in pre-defined age (1-year intervals), sex, calendar (monthly level), and socioeconomic groups.

### Socioeconomic position

We included the highest achieved educational level measured in months as an indicator of socioeconomic position by retrieving information from the Population Education Register (PER), which contains annual individual-based information about all individuals attending an education in Denmark [26]. Educational level was grouped in three: *low educational level*, i.e., no education and primary school (≤ 9 years), *medium educational level*, i.e., high school or vocational school (10–12 years), and *high educational level*, which included university and other forms advanced education (≥ 13 years). Individuals with missing information were excluded from the analyses (corresponding to 4.6% of the AMI study population; 4.4% of the COPD study population, and 4.3% of the cancer study population).

### Outcome

As most smoking-related morbidity arise from cardiovascular diseases (primarily AMI and stroke), respiratory diseases (primarily COPD), and cancers, three outcomes were included in the analyses: AMI (ICD8: 410; ICD10: I21) [27, 28], COPD (ICD8: 491–492; ICD10: J44) [29, 30], and smoking-related cancers defined as the first occurring diagnosis of cancer in bronchus and lung (ICD8: 162.1; ICD10: C34, D022), cancer in lip, mouth, oral cavity, and pharynx (ICD8: 140–149; ICD10: C00-C14, D000), and bladder cancer (ICD8: 188; ICD10: C67, D090). Analyses were conducted separately for each outcome.

### Design

We used an interrupted time series design to quantify the immediate and long-term differential impact of the Danish smoking ban across socioeconomic groups. The time periods were centered around the time of enactment of the smoking ban on the 15 August 2007, thus defining a pre- and post-ban period to be compared. The immediate impact was expressed as changes in incidence rates occurring at the time the smoking ban was implemented, whereas changes in the slope of the disease trend in the post-ban period compared with pre-ban trends determined any long-term effects of the smoking ban [31, 32]. For all outcomes we applied a pre-ban period from 1 January 2002 to 31 August 2007. However, distinct post-ban periods were defined for each outcome due to different expectations of the lag between the time of implementation of the smoking ban and subsequent effects on incidence rates, caused by differences in latency periods between diseases. For AMI and COPD, immediate and fast-acting harmful pathological changes caused by smoking or SHS exposure have been observed even at low exposure levels and the risk for cardiovascular diseases has been suggested to decrease within a few years after the elimination of exposure [33–37]. In addition, previous studies have found declining admission rates in the general population within few years after the implementation of smoke-free regulations for both outcomes [30, 38]. Consequently, we included a post-ban period of 3.3. years for AMI (end of follow- up 31 December 2010) and 5.3 years for COPD (end of follow-up 31 December 2012). We acknowledge, that the long latency period for smoking-related cancers, which involve a complex process with a slow progression of disease, makes it more difficult to study causation between the implementation of the smoking ban and subsequent changes in incidence rates. However, due to the strong causal link between active smoking or SHS exposure and risk of cancer, we argue for the importance of examining cancer rates over time. Thus, for smoking-related cancers we included the longest possible follow-up period at the time of study (8.3-years) (end of follow up 31 December 2015).

### Statistical analyses

Descriptive characteristics of the study population stratified by sex, time-period, and educational level were calculated. For each educational level we estimated age- and sex-standardized incidence rates every three months of AMI, COPD, and smoking-related cancers (European population as standard).

To examine changes in incidence rates after the smoking ban across socioeconomic groups, Poisson regression models stratified by educational level were conducted for each outcome separately. As offset, a logarithmic transformation of the follow-up time was applied. Models were fitted to estimate three parameters of interest: the underlying secular disease trend assuming to continue unaffected in absence of the smoking ban; the level change occurring in the month after the smoking ban was introduced (September 2007), expressing the immediate impact of the ban; and the post-ban change in the disease trend, estimating any long-term impact of the smoking ban when compared to pre-ban trends. Models were adjusted for age, sex, and seasonal variations, which was constrained to be the same each year and based on calendar month. The final Poisson models were defined as:

$$\mathrm{Log}(\mathrm{Y})\hspace{0.17em}=\hspace{0.17em}\upbeta_0\hspace{0.17em}+\hspace{0.17em}\upbeta _1\mathrm{Time}\hspace{0.17em}+\hspace{0.17em}\upbeta_2\mathrm{Smoking ban}\hspace{0.17em}+\hspace{0.17em}\upbeta_3(\mathrm{Time}*\mathrm{Smoking ban})\hspace{0.17em}+\hspace{0.17em}\mathrm{\beta_x}(\mathrm{Age},\mathrm{ Sex},\mathrm{ Season}),$$

where Y expresses the monthly incidence rate, *β*_*0*_ is the log-incidence rate at baseline for the outcome in question,* β*_*1*_*,* is the disease trend in the pre-ban period (continuous, linear predictor for time); *β*_*2*_*,* is the immediate level change in the rate following the implementation of the smoking ban (binary indicator variable with a value of zero in the pre-ban months and a value of 1 in the post-ban period), and* β*_*3*_*,* is the change in disease trend in the post-ban period compared to the pre-ban trend (interaction term between the linear predictor for time and the binary indicator). *β*_*x*_ denotes the effects of the covariates.

Coefficients were exponentiated to derive incidence rate ratios (IRR) for presentation of results, and trend estimates were calculated as changes in rates every third year. Further, to examine changes in the socioeconomic gradient in incidence of AMI, COPD, and smoking related cancers pre- and post-ban, Poisson regression models accounting for the underlying secular trend, sex, age, and seasonality were performed. To account for overdispersion we applied a scale parameter in all models that allowed the variance to be bigger than the mean. Analyses were performed in SAS software (version 9.4) and figures were created in STATA (version 15.1).

## Results

In both the low, medium, and high educational group, lower incidence rates of AMI were observed in the post-ban period compared to the pre-ban period for both men and women (Table 1). For COPD, lower post-ban rates, compared to pre-ban, were only observed among men with a medium educational level (pre-ban IR: 457.1, post-ban IR: 451.3) and women with a high educational level (pre-ban IR: 236.9, post-ban IR: 235.3), and for smoking-related cancers among men with a medium (pre-ban IR: 296.1, post-ban IR: 285.0) or high educational level (pre-ban IR: 218.3, post-ban IR: 196.9).

**Table 1:** Incident cases of acute myocardial infarction, chronic obstructive pulmonary disease, and smoking-related cancers, total risk time, and age-standardized incidence rates in the pre- and post-ban period by educational level and sex, Denmark

	**Pre-ban period**^a^		**Post-ban period**^b^	
	Events^c^/risktime (1000 py^e^)	IR^d^ per 100.000 py^e^	Events^c^/risktime (1000 py^e^)	IR^d^ per 100.000 py^e^
**Men**				
**Acute myocardial infarction**				
Low educational level	13,565/2513.9	580.2	7215/1407.4	510.1
Medium educational level	13,230/4067.1	503.8	7769/2479.0	435.8
High educational level	4726/2122.2	396.1	2890/1,378.1	330.4
**Chronic obstructive pulmonary disease**				
Low educational level	14,686/2553.5	593.8	14,522/2245.3	616.5
Medium educational level	11,158/4128.0	457.1	12,982/4032.4	451.3
High educational level	3013/2146.2	259.9	3712/2270.1	276.0
**Smoking-related cancers**				
Low educational level	8549/2614.5	327.3	13,099/3531.7	330.0
Medium educational level	7902/4165.4	296.1	14,491/6396.0	285.0
High educational level	2569/2153.4	218.3	4852/3654.0	196.9
**Women**				
**Acute myocardial infarction**				
Low educational level	11,438/3300.8	290.9	6196/1811.4	251.1
Medium educational level	4212/3480.0	215.8	2729/2166.9	186.6
High educational level	1541/2430.5	164.4	1170/1673.3	152.4
**Chronic obstructive pulmonary disease**				
Low educational level	19,934/3255.6	496.6	19,770/2775.5	533.7
Medium educational level	7939/3460.5	356.5	9527/3455.9	360.7
High educational level	2663/2423.1	236.9	3483/2745.7	235.3
**Smoking-related cancers**				
Low educational level	8303/3360.9	194.5	13,670/4377.4	219.3
Medium educational level	3685/3497.9	153.0	7738/5521.2	163.9
High educational level	1350/2435.4	102.0	3205/4497.8	113.2

^a^Pre-ban period: 01.01.02 to 31.08.07

^b^Post-ban period for acute myocardial infarction: 01.09.07 to 31.12.10; chronic obstructive pulmonary disease: 01.09.07 to 31.12.12; smoking-related cancers: 01.09.07 to 31.12.15

^c^Events fulfilling inclusion criteria

^d^Age-standardized incidence rate

^e^Person-years

**Fig. 1:**
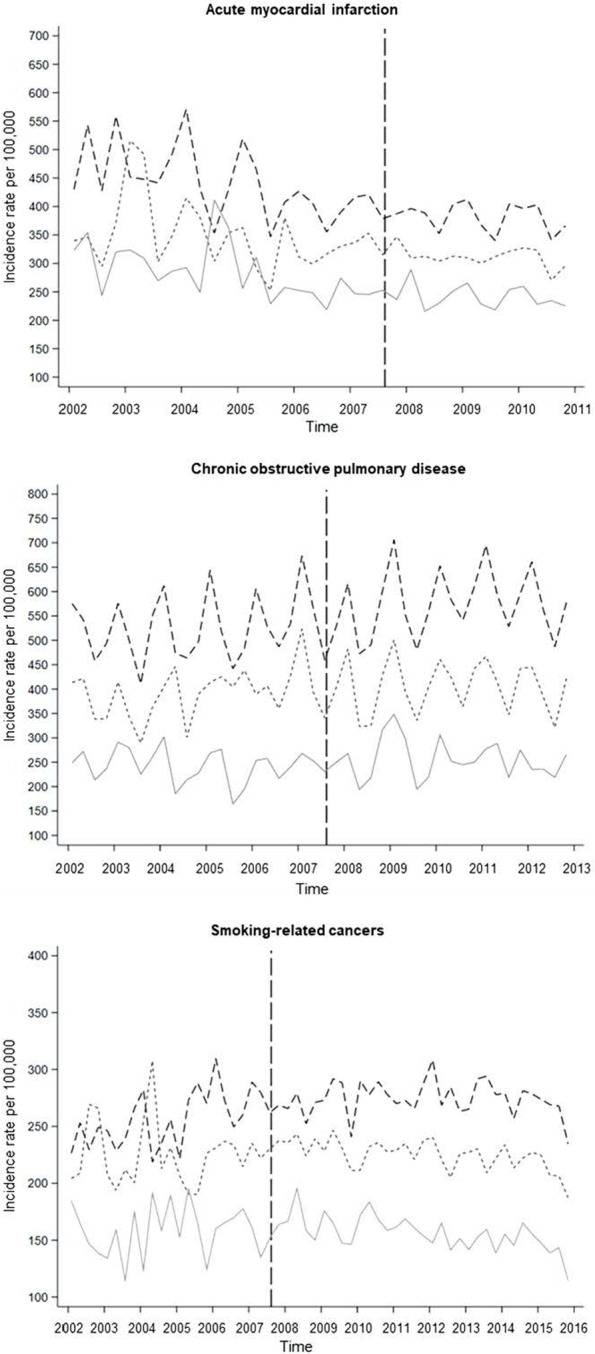
Age- and sex-standardized incidence rates every three months for acute myocardial infarction, chronic obstructive pulmonary disease, and smoking-related cancers divided by educational level. The dashed vertical line shows the time of implementation of the Danish smoking ban in 2007

Declining age- and sex-standardized incidence rates for AMI were observed during the whole study period in the low, medium, and high educational group (Fig. 1). Incidence rates for COPD and smoking-related cancers appeared approximately constant in all educational groups during the study period. A socioeconomic gradient was observed for all outcomes during the whole study period with persistent higher incidence rates among the lowest educated group. Additionally, the difference between the socioeconomic groups appeared to be constant in the pre- and post-ban period for all outcomes. Seasonal variations were observed for AMI and COPD.

No consistent post-ban changes in the disease trend were found for AMI, COPD, and smoking-related cancers across educational groups (Table 2). For AMI, estimates indicated that the disease trend every third year declined to a slightly lesser extent following the implementation of the smoking ban among individuals with low (IRR: 1.03; 95% CI: 1.01–1.04) and medium educational level (IRR: 1.03; 95% CI: 1.02–1.05). Similarly, the COPD trend appeared to decline less steeply in the post-ban period among those with a low educational level (IRR: 1.02; 95% CI: 1.01–1.02). However, estimates and absolute differences were very small.

**Table 2:** Changes in rates and trend of acute myocardial infarction, chronic obstructive pulmonary disease, and smoking-related cancers after the implementation of a national smoking ban compared with the pre-ban period, stratified on educational level, Denmark, 2002–2015

	**Pre-ban trend (IRR**^a^**)**^b,c^	**Post-ban trend (IRR**^a^**)**^b,d^	**Change in trend** **(IRR**^a^**) (95%CI)**	**Immediate level change (IRR**^a^**) (95%CI)**
**Acute myocardial infarction**				
Low educational level	0.87	0.90	1.03 (1.01–1.04)	0.95 (0.92–1.00)
Medium educational level	0.85	0.88	1.03 (1.02–1.05)	0.98 (0.94–1.03)
High educational level	0.85	0.86	1.02 (0.99–1.05)	1.03 (0.96–1.12)
**Chronic obstructive pulmonary disease**				
Low educational level	0.96	0.97	1.02 (1.01–1.02)	1.00 (0.97–1.03)
Medium educational level	0.96	0.96	1.00 (1.00–1.01)	1.01 (0.97–1.05)
High educational level	0.90	0.91	1.01 (0.99–1.02)	1.10 (1.03–1.17)
**Smoking-related cancers**				
Low educational level	1.02	1.01	0.99 (0.98–1.00)	1.01 (0.97–1.05)
Medium educational level	0.96	0.95	0.99 (0.99–1.00)	1.06 (1.02–1.11)
High educational level	0.94	0.93	1.00 (0.98–1.01)	1.09 (1.02–1.17)

^a^Incidence rate ratio

^b^Change per three years

^c^Pre-ban period: 01.01.02 to 31.08.07

^d^Post-ban period for acute myocardial infarction: 01.09.07 to 31.12.10; chronic obstructive pulmonary disease: 01.09.07 to 31.12.12; smoking-related cancers: 01.09.07 to 31.12.15

An immediate ten percent increase in incidence of COPD following the smoking ban was found among those with high educational level (IRR: 1.10; 95% CI: 1.03–1.17) (Table 2). Further, immediate increases of smoking-related cancers were observed in the medium (IRR: 1.06; 95% CI: 1.02–1.11) and high educational group (IRR: 1.09; 95% CI: 1.02–1.17). No immediate post-ban changes in rates were found for AMI.

Clear socioeconomic gradients in incidence of AMI, COPD, and smoking-related cancers were observed in both the pre- and post-ban period (Table 3). In the pre-ban period, the incidence rate of AMI was 72% higher among the low educated compared to those with high educational levels (IRR: 1.72, 95%CI: 1.67–1.77). Similar socioeconomic gradients were observed for COPD (2.45, 95%CI: 2.39–2.52) and smoking-related cancers (1.74, 95%CI: 1.68–1.80). The incidence rate ratios did not change in the post-ban period for any outcomes.

**Table 3:** Age- and sex-standardized incidence rates for acute myocardial infarction, chronic obstructive pulmonary disease, and smoking-related cancers by educational level and time period, Denmark, 2002–2015

	**Pre-ban period**^a^		**Post-ban period**^b^	
	IR^c^ (100.000 py^d^)	IRR^e^ (95%CI)	IR^c^ (100.000 py^d^)	IRR^e^ (95%CI)
**Acute myocardial infarction**				
Low educational level	435.5	1.72 (1.67–1.77)	380.6	1.71 (1.65–1.77)
Medium educational level	359.8	1.42 (1.38–1.46)	311.2	1.39 (1.34–1.44)
High educational level	280.2	ref	241.4	ref
**Chronic obstructive pulmonary disease**				
Low educational level	545.2	2.45 (2.39–2.52)	575.1	2.45 (2.39–2.52)
Medium educational level	406.8	1.75 (1.70–1.80)	406.0	1.75 (1.70–1.80)
High educational level	248.4	ref	255.7	ref
**Smoking-related cancers**				
Low educational level	260.9	1.74 (1.68–1.80)	274.6	1.79 (1.75–1.84)
Medium educational level	224.6	1.50 (1.45–1.55)	224.5	1.53 (1.49–1.57)
High educational level	160.1	ref	155.0	ref

^a^Pre-ban period: 01.01.02 to 31.08.07

^b^Post-ban period for acute myocardial infarction: 01.09.07 to 31.12.10; chronic obstructive pulmonary disease: 01.09.07 to 31.12.12; smoking-related cancers: 01.09.07 to 31.12.15

^c^Age- and sex-standardized incidence rate

^d^Person years

^e^Incidence rate ratio

## Discussion

In this nationwide study including the entire Danish adult population, no differential impact of the Danish national smoking ban from 2007 on incidence of AMI, COPD, and smoking-related cancers was found across socioeconomic groups. Neither immediate changes in incidence rates nor long-term changes in the disease trend were observed for any outcomes in the years after the smoking ban compared to pre-ban levels. The social gradient observed in the pre-ban period continued at the same level post-ban.

Overall, previous studies examining the contribution of smoking bans to reduce socioeconomic inequalities have primarily focused on post-ban changes in smoking behavior, such as smoking prevalence, SHS exposure, and cessation rates [6, 17, 39, 40]. Two systematic reviews found insufficient evidence that restrictions in workplaces and public places are more effective in reducing smoking in higher socioeconomic groups [6, 17]. Hill et al. found higher workplace SHS exposure among socioeconomic disadvantaged groups, but no clear evidence of how smoking restrictions affect this gradient since studies have failed to demonstrate differential effects [6]. In addition, no educational differences in successful smoking cessation were observed after a smoking ban in the hospitality industry in the Netherlands [40]. Lastly, Federico et al. suggested that the immediate 1.6% decrease in smoking prevalence and 4.5% increase in smoking cessation following the Italian smoking ban, which was observed only among low educated women, subsequently reversed over time [41].

Previous studies examining the differential impact of smoking bans on smoking-related morbidity are limited. Contrary to the present findings, a study examining COPD admissions following two succeeding smoking bans in Spain suggested greater declines of COPD in provinces with lower levels of socioeconomic development [21]. Further, Cesaroni et al. observed reductions in acute coronary events in the population of Rome after smoking was banned in all public places, with the largest reduction among those living in low socioeconomic census blocks [22]. Socioeconomic differences were also found in mortality of ischemic heart disease and COPD following the national Irish smoking ban, where health benefits were concentrated among the most disadvantaged groups [7].

We did not find any differential effects across socioeconomic groups, which might be explained by differences between countries in standards of living, available health care systems as well as population smoking rates. Another explanation may be that the Danish smoking ban was considerably less comprehensive than those implemented in other countries [16, 42]. Previous studies have shown that comprehensive smoking bans are more effective in reducing several adverse health outcomes such as AMI, COPD, and lung cancer compared to partial bans [29, 43, 44]. Further, comprehensive bans have been found to enhance public support and compliance with regulations and are associated with more quit-attempts and subsequent quit-success compared to partial bans [45, 46]. The Danish smoking ban comprised several exemptions such as allowing smoking in bars under 40m^2^ where no food is served, in one-man offices, in commercial vehicles, and in mental health care services [16]. This has probably influenced the lack of a measurable impact of the ban.

Two main pathways may link smoking bans to declines in disease rates, including directly reduce SHS exposure and indirectly reduce smoking prevalence by encourage quitting and prevent initiation [47]. Previous research demonstrates that comprehensive smoking bans are effective in reducing SHS exposure, while inconsistent evidence exists for smoking prevalence [4].

The smoking prevalence in Denmark has steadily decreased with approximately one percentage point each year from 2000 to 2012 where a stagnation followed [48]. Thus, no accelerated decrease in the smoking prevalence occurred in the years after the smoking ban [28, 49]. The exposure to SHS in workplaces, educational institutions, and public settings declined in the years preceding the smoking ban and continued to decrease post-ban [49]. A survey from 2009 showed that self-reported daily SHS exposure in workplaces and educational institutions decreased substantially from 27% in 2004 and 21% in 2006 to 5% in 2009 [49]. We found no changes in disease trends post-ban in any socioeconomic groups, thus the reduction in SHS exposure following the smoking ban is not reflected in our findings. Further, attributing the decrease in SHS exposure solely to the implementation of the smoking ban must be done with caution.

Smoking bans may also influence smoking behavior through a decrease in the social acceptability of smoking and changes in social norms [50–52]. In the years prior to the ban, public information announcing the impending changes, brought out an extensive media attention on the Danish smoking legislation, which increased the negative public awareness on smoking [28, 53]. In addition, individual-level interventions such as smoking cessation programs or voluntary workplace regulations were introduced throughout Denmark in these years. Two years before the enactment, 35% had introduced local smoking regulations totally banning indoor smoking in workplaces and educational institutions [49]. This increased to 48% in 2006 and 67% in 2007. Thus, a considerable part of the Danish population was already covered by local smoking restrictions at the time of the national smoking ban. The influence of these contextual factors associated with the smoking ban may have limited the direct measurable impact of the ban.

Limitations of this study include the expected time from onset of the smoking ban to manifestation of disease, which vary between diseases and in people with different magnitudes of risk. Especially, the latency period for smoking-related cancers is quite extensive. Thus, the results related to smoking-related cancers are considered preliminary. Including a longer follow-up period could have considered the possible slow responsiveness to behavioral changes among smokers caused by smoking addiction. However, increasing the period for each outcome would induce uncertainties about whether any changes in disease rates could be linked to the implementation of the smoking ban. We do not believe that a increasing the length of the post-ban period would change the findings.

This study has several strengths. First, the study is based on high quality data from nationwide registers, which enabled the inclusion of a large sample size providing great statistical power to the analyses. Combined with the detailed individual-level linkage between registers, we were able to detect even small variations in disease trends. The linkage to the RCD allowed us to include acute fatal events outside hospital, which provided a more complete measure of disease incidence. As the registers are continuously updated, thus providing many data points over time, the pre-ban trend was estimated with precision, and long follow up periods were applied. Further, because all residents in Denmark are entitled to publicly financed healthcare and we included the whole Danish adult population, the risk of selection bias was eliminated. In the analysis, we accounted for seasonality and the underlying secular disease trend which strengthened the findings.

We defined socioeconomic position as the individual highest achieved education. Research suggests that education represents a valid indicator of socioeconomic position as it often determines future employment and income and is relatively stable over an adult life span [54, 55]. Through the PER we were able to quantify the measurement comparatively accurate.

Overall, national health targets stand little chance of being reached without specific attention to the health of the socioeconomic disadvantaged groups [10]. Tobacco control policies may have considerable potential to reduce smoking inequalities, especially those targeting pricing on tobacco products, tobacco advertising, and subsidized smoking cessation services [18]. Preferably, implementing multiple policies as a part of a comprehensive tobacco control approach could be prioritized as initiatives individually may affect different sociodemographic groups. Understanding these nuances is important for creating more robust tobacco control policies beneficial for all population subgroups.

## Conclusion

We found no evidence of a differential impact of the national smoking ban from 2007 on three smoking-related diseases in the Danish adult population. Neither immediate nor long-term changes in incidence of any outcome were observed post-ban compared to pre-ban levels across socioeconomic groups. Future tobacco control measures in Denmark should consider which measures most effectively targets low socioeconomic groups to decrease the current strong socioeconomic inequality in health.

## Data Availability

Data used in this study are available upon request to Statistics Denmark.

## Abbreviations

SHS: Second-hand smoke
COPD: Chronic obstructive pulmonary disease
AMI: Acute myocardial infarction
ICD: International Classification of Disease
PER: Population Education Register
